# Trametinib therapy for children with neurofibromatosis type 1 and life‐threatening plexiform neurofibroma or treatment‐refractory low‐grade glioma

**DOI:** 10.1002/cam4.3910

**Published:** 2021-05-03

**Authors:** Rebecca Ronsley, Celine D. Hounjet, Sylvia Cheng, Shahrad Rod Rassekh, Walter J. Duncan, Christopher Dunham, Jane Gardiner, Arvindera Ghag, Jeffrey P. Ludemann, David Wensley, Wingfield Rehmus, Michael A. Sargent, Juliette Hukin

**Affiliations:** ^1^ Division of Hematology, Oncology & BMT Department of Pediatrics University of British Columbia Vancouver Canada; ^2^ Faculty of Medicine University of British Columbia Vancouver Canada; ^3^ Division of Pediatric Cardiology Department of Pediatrics University of British Columbia Vancouver Canada; ^4^ Division of Anatomic Pathology Department of Pathology University of British Columbia Vancouver Canada; ^5^ Division of Pediatric Ophthalmology Department of Surgery University of British Columbia Vancouver Canada; ^6^ Division of Pediatric Orthopedic Surgery Department of Surgery University of British Columbia Vancouver Canada; ^7^ Division of Pediatric Otolaryngology Department of Surgery University of British Columbia Vancouver Canada; ^8^ Division of Pediatric Respiratory Medicine Department of Pediatrics University of British Columbia Vancouver Canada; ^9^ Division of Dermatology Department of Pediatrics University of British Columbia Vancouver Canada; ^10^ Division of Pediatric Neuro‐Radiology Department of Radiology University of British Columbia Vancouver Canada; ^11^ Division of Neurology Department of Pediatrics University of British Columbia Vancouver Canada

**Keywords:** low‐grade glioma, neurofibromatosis, pediatric, plexiform neurofibroma, trametinib

## Abstract

**Purpose:**

To describe a series of children with extensive PNF or treatment refractory PLGG treated on a compassionate basis with trametinib.

**Methods:**

We report on six patients with NF‐1 treated with trametinib on a compassionate basis at British Columbia Children's Hospital since 2017. Data were collected retrospectively from the patient record. RAPNO and volumetric criteria were used to evaluate the response of intracranial and extracranial lesions, respectively.

**Results:**

Subjects were 21 months to 14 years old at the time of initiation of trametinib therapy and 3/6 subjects are male. Duration of therapy was 4–28 months at the time of this report. All patients had partial response or were stable on analysis. Two patients with life‐threatening PNF had a partial radiographic response in tandem with significant clinical improvement and developmental catch up. One subject discontinued therapy after 6 months due to paronychia and inadequate response. The most common adverse effect (AE) was grade 1–2 paronychia or dermatitis in 5/6 patients. There were no grade 3 or 4 AEs. At the time of this report, five patients remain on therapy.

**Conclusion:**

Trametinib is an effective therapy for advanced PNF and refractory PLGG in patients with NF‐1 and is well tolerated in children. Further data and clinical trials are required to assess tolerance, efficacy and durability of response, and length of treatment required in such patients.

## Graphical abstract

Here, we report on response to Trametinib, a MEK inhibitor in children with NF‐1. Our experience presented in this report demonstrates that Trametinib is an effective therapy for advanced, life‐threatening PNF, and treatment‐refractory PLGG in patients with NF‐1 and is well tolerated in children.

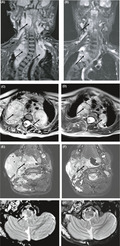

## 1 INTRODUCTION

Plexiform neurofibromas (PNF) are benign nerve sheath tumors occurring in neurofibromatosis type 1 (NF‐1). Their growth during childhood may cause refractory pain, neurological deficits, organ dysfunction due to compression, cosmetic issues or deformity, and rarely mortality.^1^, ^2^ Historically, treatment of PNF has been limited to repeated debulking surgery and ineffective medical therapy.^3^, ^4^, ^5^

The mainstay of treatment for unresectable pediatric low‐grade gliomas (PLGG) in NF‐1 is chemotherapy. A variety of conventional chemotherapeutic options have been used with variable responses, and up to 30% progress by 5 years off therapy.^6^, ^7^, ^8^

In NF‐1, there is a lack of functional neurofibromin resulting in a dysregulation of RAS/mitogen‐activated protein kinase (MAPK) pathways and up to 50% of patients will develop an intracranial PLGG or a PNF.^9^, ^10^ Recent research has implicated a dysregulation of the MEK signaling system^11^ downstream of RAS in both PLGGs and PNF, indicating a potential therapeutic opportunity for targeting this pathway.^12^, ^13^

Subsequent to RAS pathway‐targeted therapy trials, MEK inhibition has been explored in the treatment of patients with extensive PNF with NF‐1. In phase 1 and phase 2 clinical trials in this group, selumetinib was used in pediatric patients for treatment of otherwise inoperable PNF. Shrinkage of the PNF was observed in 70% of patients. In addition, many patients experienced alleviation of pain and improvement in function.^14^ Similarly, Phase 1 and Phase 2 clinical data of selumetinib in refractory PLGG is encouraging, demonstrating a sustained partial response in up to 40% of children with these tumors.^15^, ^16^

Trametinib is a reversible highly selective inhibitor of MEK1/2 activation and kinase activity. Phase 1 and pre‐clinical data demonstrate promising results in a small number of children with PNF or optic pathway gliomas, available in conference proceedings format only to date.^17^, ^18^, ^19^ Furthermore, tolerance and efficacy of trametinib has been shown^20^, ^21^, ^22^ in a small number of children in the setting of LGG and melanoma without NF‐1. Thus, trametinib has become an attractive consideration for children with symptomatic PNF and refractory progressive PLGG; there is currently a phase 2 Canadian clinical trial evaluating trametinib in children with LGG and PNF.^23^ Given this promising early data, and lack of effective alternative options, trametinib was used at our center on a compassionate access basis, for six pediatric patients with neurofibromatosis type 1 and either progressive PNF or refractory LGG.

## 2 METHODS

### 2.1 Study design

Following approval by the University of British Columbia Clinical Research Ethics Board, a retrospective review was conducted on six patients with NF‐1 <20 years of age with a PLGG or severe plexiform neurofibroma treated with trametinib on a compassionate basis at British Columbia Children's Hospital between December 2017 and May 2020.

### 2.2 Treatment protocol

Trametinib dosing was based on phase 1 safety data in pediatric patients.^24^ Three of the six patients received a dose of 0.025 mg/kg/day, and two patients age <6 years were increased to 0.032 mg/kg/day. One patient received a dose of 0.016 mg/kg/day. Patient 3 received low‐dose therapy due to previous history of retinal edema on exposure to trametinib at another center. Patients 1 and 2 were started at standard dosing 0.025 mg/kg based on information available at the time, when more information became available regarding safety of higher dosing in the child under 6 years of age, these patients were switched to the standard dose for age of 0.032 mg/kg. Patient 5 was started at a slightly lower dose to begin with due to tablet size and for convenience. This was rounded up as his weight increased to 0.025 mg/kg. One patient received the oral solution format of trametinib via g‐tube provided through the Novartis special access program. Patients were monitored regularly for adverse effects (AEs) by the oncology, cardiology, and ophthalmology services.

### 2.3 Variable definitions and analysis

Patient records were reviewed including diagnosis, indication, trametinib dosing, imaging, and adverse events.

Radiologic response of gliomas was analyzed by a single neuroradiologist using Response Assessment in Pediatric Neuro‐oncology (RAPNO) criteria; all MRI scans used for RAPNO analysis were axial T2/FLAIR/T1 contrast or in the plane where measurements were most reproducible.^25^ Neurofibromas were analyzed using the volumetric analysis; all volumetric analyses were conducted on axial T2 or STIR MRI scans.^26^, ^27^ For RAPNO analysis, minor response was 25%–49% shrinkage, partial response was 50% or greater decrease of all measurable T1 contrast/T2/FLAIR LGG; progression was defined as 25% or more increase in T1 contrast/T2/FLAIR LGG lesions. For volumetric analysis, partial response >20% decrease, progression >20% increase. AEs were collected from all clinic visit documentation during trametinib treatment and were graded using Common Terminology Criteria for AEs version 5 (CTCAEv5).^28^

## 3 RESULTS

### 3.1 Patient characteristics

Six patients with NF‐1 were treated for PLGGs or PNF with trametinib on a compassionate basis between December 2017 and May 2020. Patient demographics and trametinib indication and treatment are presented in Table 1. Median age was 9 years (range 1–14 years) at start of trametinib therapy. Five of six patients including all patients with PLGG had progression on chemotherapy treatments prior to trametinib. Patients received trametinib for an average period of 12.17 months (range 4–28 months). Five of six patients remain on trametinib therapy. Trametinib was stopped due to AEs in one patient. All patients are alive at the time of this report.

**TABLE 1:** Patient Characteristics

Patients	Age^a^	Sex	NF1	Prior therapy	Trametinib Indication	Duration of trametinib (months)	Dosage (mg/kg)	Current Therapy Status	Clinical Changes
1	4 yo	F	Yes	CBD oil, turmeric	Progressive PNF of neck and face, critical airway, obstructive sleep apnea, hearing loss, and speech delay	16	0.025 (12 mos), 0.032 (4 mos)	Ongoing	Improved hearing and language, and cosmesis Resolution of CPAP need
2	21 mo	F	Yes	Imatinib	Progressive PNF of neck, thorax, abdomen, sleep apnea, dysphagia, failure to thrive, hearing loss, developmental delay, and severe scoliosis	28	0.025 (12 mos), 0.032 (16 mos)	Ongoing	Improved hearing and cosmesis Resolution of FTT and dysphagia Resolution of hypertension, resolution of BiPAP need, improved development
3	11 yo	F	Yes	Various chemotherapies^b^	Optic pathway glioma with worsening vision loss	8	0.016	Ongoing	Vision improved 0.1 logmar in each eye, improved headaches
4	14 yo	M	Yes	VCR and CBP	Optic chiasmic/hypothalamic glioma with vision loss, and PNF of foot with weakness	4	0.025	Ongoing	Vision improved 0.1 logmar in each eye
5	9 yo	M	Yes	VCR and CBP, bevacizumab plus irinotecan	Hypothalamic/chiasmatic glioma, vision loss, hypothyroidism, and growth hormone deficiency	13	0.019 (4 mos), 0.025 (9 mos)	Ongoing	Stable
6	14 yo	M	Yes	Surgical excision, interferon, imatinib, and irbesartan	PNFs of lumbosacral plexus and legs, hip dislocation, scoliosis, and leg weakness and pain	4	0.025	Stopped due to paronychia	Decrease in pain and decrease in size of left thigh, improved mobility

Abbreviations: CBD, cannabidiol; CBP, carboplatin; FTT, failure to thrive; PNF, plexiform neurofibroma; VBL, vinblastine; VCR, vincristine.

^a^ At trametinib initiation.

^b^ Including: VCR and CBP; VBL; VCR, cyclophosphamide and cisplatin, bevacizumab and irinotecan; VCR and dactinomycin; trametinib.

### 3.2 Indications for trametinib use

**FIGURE 1:**
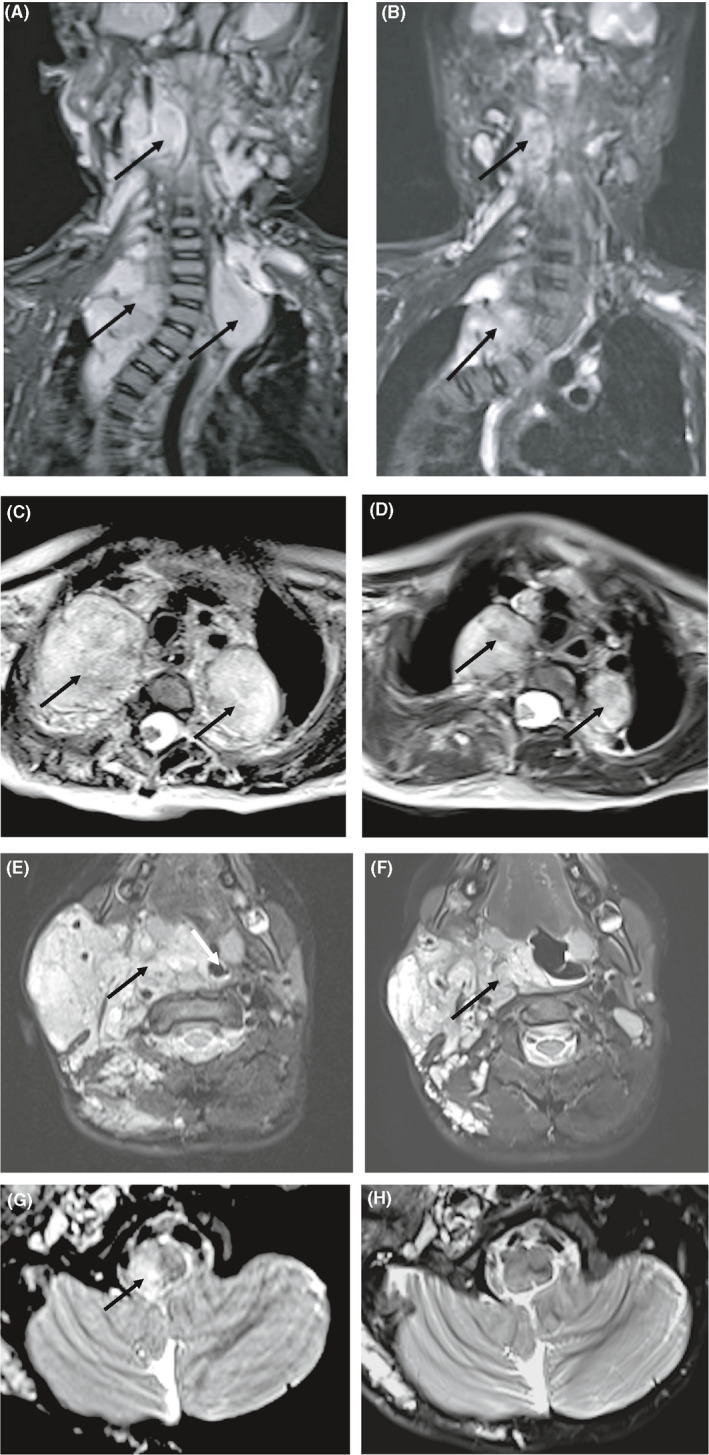
Radiographic Change During Trametinib Therapy. *Black arrow indicates lesion. (A) Case 2 – Coronal STIR image in a 21 month old female with progressive neurofibromas of neck and mediastinum prior to trametinib therapy. (B) Case 2‐ Radiographic improvement (volumetric partial response) after 26 months therapy with trametinib. (C) Case 2‐ Axial T2 image demonstrates the bilateral posterior mediastinal masses prior to trametinib. (D) Case 2‐ Radiographic improvement (volumetric partial response) after 26 months therapy with trametinib. (E) Case 1‐ Axial fat‐saturated T2 image in a 4 year old female with progressive neurofibromas of the face prior to trametinib. There is significant displacement and narrowing of the nasopharyngeal airway. White arrow indicates the nasopharyngeal airway. (F) Case 1‐ Radiographic improvement (volumetric partial response) after 17 months therapy with trametinib. A laryngeal mask airway was used for this sedated MRI and distends the displaced nasopharyngeal airway. (G) Case 1‐ Axial fat‐saturated T2 image demonstrates a low grade glioma of the right posterior medulla prior to trametinib therapy. (H) Case 1‐Improvement of size and signal of the low grade glioma after 17 months therapy with trametinib (RAPNO partial response)

Trametinib use in two patients was due to life‐threatening respiratory compromise resulting from airway compression by PNF, requiring assisted ventilation during sleep (Figure 1A and B). Both also had cosmetic issues, hearing loss, and language delay, and one had oral feeding intolerance with dysphagia, vomiting, aspiration, global developmental delay, and failure to thrive (FTT), as well as severe scoliosis secondary to the PNF. Three of six patients had gliomas involving the optic chiasm and hypothalamus associated with endocrine dysfunction and decline in vision. One of which also had a PNF in the dorsum of left foot with associated weakness. The remaining patient has extensive PNF causing scoliosis, hip dislocation and fracture, leg deformity, leg‐length discrepancy, and weakness.

### 3.3 Radiologic response

**FIGURE 2:**
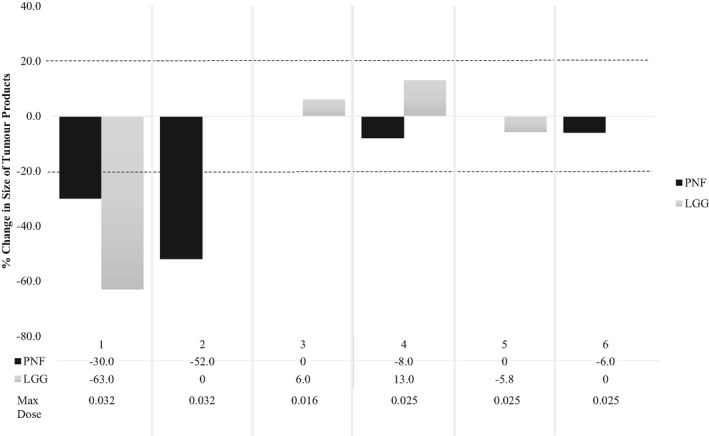
Radiologic response following trametinib therapy

Radiologic response by subject is presented in Figure 2. On RAPNO analysis of intracranial lesions, one lesion demonstrated partial response and three were stable. On volumetric analysis of PNF, two had a gradual partial response and two patients were stable. Overall, all subjects’ lesions demonstrated stable or improved response since initiation of trametinib therapy.

### 3.4 Clinical changes

Clinical changes observed following trametinib therapy are shown in Table 1. Life‐threatening respiratory compromise secondary to large PNF improved in both patients such that both patients no longer require any respiratory support. As well, both of these patients are now able to tolerate solid foods and thickened fluids orally with marked developmental catch up. Both have resolution of conductive hearing loss; specifically, prior to therapy, one patient had bilateral conductive moderate hearing loss from 500–5000 Hz to 50 dB and the other had right ear conductive moderate hearing loss from 250–8000 Hz down to 50 dB and both patients now have normal hearing. Two patients with a PLGG have improved visual function and one improvement of associated headaches. Two patients experienced a reduction in pain, and one retrospectively commented that he had experienced a decrease in size of the thigh deformity while treated with trametinib. This change was only described to our team after discontinuation of therapy. This patient subsequently noted worse pain and thigh girth re‐enlargement after trametinib was stopped.

### 3.5 Adverse effects

Treatment was complicated by grades 1 and 2 adverse effects AEs including abdominal cramping, paronychia, atopic dermatitis, folliculitis, aphthous ulcers, chondrodermatitis nodularis helicis, headache, and pneumonia. The most common AEs were paronychia and atopic dermatitis, experienced by five of six patients. Two patients required intermittent oral antibiotics for paronychia (grade 2), one of whom stopped trametinib because of paronychia discomfort. One of these patients had paronychia prior to start of trametinib, but due to the life‐threatening nature of the disease, the trametinib was started. One patient experienced grade 2 acne requiring doxycycline and isotretinoin in addition to topical agents. No patient developed cardiac, ophthalmologic, or growth plate toxicity.

## 4 DISCUSSION

We present a series of six pediatric patients with NF‐1 treated with trametinib for large, symptomatic, progressive PNFs, or refractory progressive symptomatic optic pathway gliomas. These cases demonstrate the utility of trametinib in the setting of life‐threatening lesions in children with NF‐1.

In this series, two patients had a partial response of the extensive plexiform neurofibroma tumor burden with associated improvement in dysfunction, one of them also had a minor response of an asymptomatic PLGG. The remainder of the patients had stable disease on imaging with three demonstrating mild clinical improvement. For those with tumor shrinkage, there was a gradual reduction in tumor volume on serial imaging and improvement in clinical status. RAPNO and volumetric analysis criteria were used to systematically analyze response in this series, according to the most recent consensus for the evaluation of PLGG^25^ and PNF.^24^, ^26^, ^27^ Prior to this series, six children with PLGG were treated with trametinib following progression on conventional therapy and were described retrospectively. The authors reported two partial responses and three minor responses, with one progression after a median duration of 11 months.^35^ In another single institution experience, trametinib was used for recurrent or progressive PLGG; after a median duration of 13 months, one patient had a partial response, one had a minor response, and five had stable disease.^36^ Four out of 10 patients in that series had NF‐1, of which two had a partial response and two had stable disease. Two pediatric cases were also described by Miller et al, where trametinib was used to treat inoperable pilocytic astrocytomas.^21^ Similar to our series, those two cases were also heavily pre‐treated with chemotherapy and both cases had a gradual reduction in tumor size. In our cohort stabilization of the optic pathway, glioma also accompanied stabilization or improvement in visual impairment. By comparison, a phase 1 and 2 studies evaluated selumetinib (another MEK inhibitor) in children with PLGG and the authors observed a partial response after 13 cycles in 36%–39% of patients.^15^, ^16^ Selumetinib is not yet available for patients in Canada and therefore trametinib provides a reasonable alternative consideration for refractory LGG, in particular in the setting of NF‐1.

The most dramatic benefit in our series was the response to trametinib in the setting of life‐threatening PNF. In conjunction the functional improvement has been dramatic: such that neither require ongoing respiratory support, both have thrived one no longer requiring tube feeds, both have improved hearing and made significant developmental progress. Prior to the phase 1 data of selumetinib,^14^ options for mitigating this disease were very limited. Our data demonstrates that trametinib can induce reduction of PNF tumor burden and improved function and cosmesis in young children with progressive, symptomatic disease. Prior to our series, there is only one published case report of PNF treated with trametinib, in which the authors describe a 22% reduction in a large PNF of the neck in an 11‐year‐old child with NF‐1.^29^ Our teenager with severe morbidity due to his extensive PNF burden had only mild clinical benefit over 6 months and was discouraged by the limited benefit and paronychia. This particular case highlights that severe deformity due to many years of progressive disease may be more difficult to reverse in older children and may require a longer duration of therapy to demonstrate benefit.

In addition to lesion stability and improvement in clinical symptoms, it is also encouraging that trametinib was well tolerated in these six cases and only one patient discontinued therapy due to an AE. Prior to this series, trametinib has been associated with skin changes, cutaneous malignancy, thromboembolism, cardiomyopathy, and ocular toxicity in adult patients.^30^, ^31^, ^32^, ^33^ In this series, the most common reported AE was paronychia, which is consistent with previous reports of trametinib in pediatric patients.^34^, ^35^, ^36^ In this series, skin side effects were managed with supportive care in the outpatient setting. Unfortunately, in one teenage patient, paronychia created discomfort and ultimately resulted in discontinuation of trametinib therapy. In retrospect, this patient noted a mild reduction in his PNF on therapy, allowing him to wear a larger variety of clothing, improved function, and reduced pain. No severe adverse events were seen; however, one patient was previously treated with trametinib at another center, and discontinued therapy due to retinal edema. Interestingly, upon transfer to our center, we initiated trametinib therapy at a reduced dose and noted a decrease in lesion size with no reported AEs or recurrence of retinal edema. Since previous reports included reversible left ventricular cardiac dysfunction during trametinib therapy,^33^ our patients underwent echocardiogram monitoring; however, cardiac dysfunction was not observed in any of these six cases.

Five of six patients in this cohort remain on therapy with continued response, and the optimal duration of therapy is not understood. Despite the prior series and current data in this report, the literature remains limited and there are many unanswered questions regarding the utility and efficacy of trametinib therapy. Currently, we do not have data to understand the duration and durability of response to trametinib in this population and to understand the potential for resistance. Although labor intensive, volumetric analyses were completed as a more accurate evaluation of these complex PNF.^27^ A strength of the analysis of our cases is that it was conducted by a single radiologist for all cases.

The two young children with developmental delay have demonstrated developmental catch up, which is likely multifactorial. It remains to be elucidated whether early MEK inhibition will have a positive effect on neurodevelopment in patients with NF‐1.^37^ Unfortunately, long‐term effects of this medication are not known when used in such young patients. In adults, cardiac toxicity has been seen which may have long‐term implications for cardiac function; however, it is worth noting that none of the patients in this series had cardiac AEs. An additional concern is the potential effect on the growth plates and long bones in children as dose‐related thickening of the growth plate and degeneration in long bones have been reported in animal studies. X‐ray evaluation of growth plates did not identify abnormality in our series. Certainly, a limitation with the use of this medication in children is the lack of understanding of late effects in this population, which should be evaluated in future clinical trials.

All of the cases presented here were children with known NF‐1 and it is encouraging that all demonstrated stable disease or response to trametinib therapy. Further large‐scale studies are needed to better understand the treatment effect of trametinib and define optimal treatment duration. Recently, a multicenter phase 2 clinical trial evaluating trametinib in pediatric patients with PNF and PLGG was opened and is currently underway in Canada.^23^, ^34^ Data from this trial will aid in establishing appropriate duration of treatment, durability of response, development of resistance, and whether there is any impact on cognition in patients with NF‐1.

## 5 CONCLUSIONS

Here, we demonstrate six pediatric cases of PNF or PLGGs and response to trametinib therapy. All of these cases had stable disease or reduction in tumor volume some with improvement of function. The most dramatic clinical benefit was in the two youngest patients with life‐threatening symptomatic PNF disease. Trametinib was well tolerated in the outpatient setting and may provide an attractive option for otherwise treatment‐refractory PLGG and symptomatic PNFs, particularly in the setting of NF‐1. This data supports further investigation into the use of trametinib in PLGG or PNF in the setting of clinical trials.

## LAY SUMMARY

Plexiform neurofibromas (PNF) and low‐grade gliomas (LGG) are benign tumors associated with Neurofibromatosis type 1 (NF‐1). There are limited options for treating extensive PNF and LGG. In this series, we describe encouraging response to Trametinib (a drug that targets a signaling pathway upregulated in NF‐1‐related tumors) in six children with treatment refractory LGG or life‐threatening PNF. All of these patients had stability or reduction of their tumor on therapy.

## PRECIS

Trametinib is an effective therapy for advanced, life‐threatening PNF, and refractory PLGG in patients with NF‐1 and is well tolerated in children.

## CONFLICT OF INTEREST

The authors have no relevant conflict of interest to disclose with respect to this work.

## ETHICS STATEMENT

This work was approved by the University of British Columbia Clinical Research Ethics Boards.

## Data Availability

Data are available upon request to the corresponding author.
